# Abacavir versus Zidovudine-based regimens for treatment of HIV-infected children in resource limited settings: a retrospective cohort study

**DOI:** 10.1186/s12887-020-1995-4

**Published:** 2020-03-03

**Authors:** Teshale Ayele Mega, Firehiwot Belayneh Usamo, Getandale Zeleke Negera

**Affiliations:** 1 Department of clinical pharmacy, School of Pharmacy, Institute of Health Sciences, Jimma University, Jimma, Ethiopia; 2 Department of Pharmacy, College of Medicine and Health Science, Dilla University, Dilla, Ethiopia

**Keywords:** HIV, Abacavir, Zidovudine, Clinical outcome, Jimma medical Centre, Ethiopia

## Abstract

**Background:**

Abacavir (ABC) and Zidovudine (AZT) based regimens are the preferred first line nucleoside reverse transcriptase (NRTIs) backbones being widely utilized for managing HIV infection in children. However, there is a dearth of data regarding the clinical outcomes and associated risk factors in Ethiopia. We compared the proportion of mortality and the rate of occurrence of Opportunistic Infections (OIs) with ABC versus AZT -based regimens in a cohort of HIV-infected children.

**Methods:**

A 42 months retrospective cohort study was conducted. A total of 179 records were reviewed by including data from October 2014 to April 2017. Data were collected on socio-demographic, clinical characteristics of patients and drug related variables. Data were analyzed using STATA13.1. Kaplan-Meier and Cox regression were used to compare survival experience and identify independent predictors. Propensity score matching analysis was conducted to elucidate the average treatment effects of each regimen over OIs.

**Result:**

Of 179 patients, 98 (54.7%) were females. The mean (+SD) age of the study subjects was 6.53 ± 2.83 years. Through 42 months analysis, a total of 4 patients (1 (1.14%) from ABC group and 3 (3.3%) from AZT group (*p* = 0.339)) were died. The incidence of opportunistic infections attributed to ABC group was 8.77/100,000 person years (py) and that of AZT was 6.9/100,000py. The incidence rate ratio (IRR) for OIs was (IRR = 0.87, 95% CI [0.49–1.53] (*p* = 0.304). Baseline CD4 count (AHR = 0.99, 95% CI [0.98–0.99]), Severe acute malnutrition (AHR = 15.92, 95% CI [5.34–47.50]), and exposure to tuberculosis treatment (AHR = 2.93, 95% CI [1.39–6.17]) were the independent predictors for the development of OIs.

**Conclusion:**

ABC and AZT based ART regimens seem to have comparable survival benefit among HIV-infected children in Ethiopia. Therefore, both regimens might be used as an alternative in resource limited settings.

## Background

Globally, an estimated 2.4 million children are living with Human Immune Virus (HIV) and 91% of them are lived in Sub-Saharan Africa [1, 2]. Ethiopia has the largest populations of HIV-infected children in the region. According to an estimate by the Federal HIV/AIDS Prevention and Control Office (FHAPCO), there are over 738,976 people living with HIV/AIDS in Ethiopia [3]. Of these, 178,500 are children younger than 15 years of age [4].

The introduction of Antiretroviral Therapy (ART) had resulted in dramatical reduction of HIV associated morbidity and mortality in children [5, 6]. However, less than 25% of those needing ART were receiving it in Ethiopia [4].

Initially, a fixed-dose combination (FDC) of Stavudine (d4T), Lamivudine (3TC) and Nevirapine (NVP) was used for the treatment of children [7]. But in 2010, the World Health Organization (WHO) guideline replaced d4T with ABC due to toxicity concerns [8]. The current ART guidelines recommends 3TC with either ABC or AZT as the preferred nucleoside reverse-transcriptase inhibitors (NRTIs) backbone for children [9]. Tenofovir (TDF) is not recommended by WHO in those younger than 10 years, because of its long-term effects on bone metabolism and renal function [10]. Zidovudine is associated with bone marrow suppression, particular in malnourished children [11]. Whereas ABC is linked with hypersensitivity reactions, especially in patients who are positive for the HLA-B*5701 allele, although these are rare in Africa because of a lower risk-allele prevalence [12, 13].

A wide range of randomized clinical trials (RCTs) are conducted to provide a robust evidence base for the treatment of adults with combination of ART [14–17]. In contrast, studies comparing different ART combinations are few in children. Although, the available studies demonstrated comparable or greater efficacy and safety of ABC compared with AZT in combination therapy regarding virological response and adverse effect [11, 18, 19], the effect of ABC on survival and factors influencing the occurrence of OIs and mortality in low income nations are rarely evaluated.

After 2 years of 2010 WHO guideline recommendation, Ethiopia implemented the d4T phase-out program and ABC becomes routinely utilized in the current practice setup since 2012 [20]. However, there is no study comparing the relative efficacy of ABC and AZT in HIV-infected children in Ethiopia. A recent study conducted in South Africa reported that children who were on ABC based regimen had lower proportion of death compared with those on d4T, even though it’s not statistically significant [21]. In this study, we compared the clinical outcomes of ABC versus AZT in combination with 3TC in terms of their clinical effects and associated risk factors in a cohort of HIV-infected Ethiopian children.

## Methods

### Study design and setting

A retrospective cohort study was conducted among 179 HIV-infected children in Jimma Medical Center (JMC). The study was conducted from April 10 to May 10, 2017 by including data from October 2014 to April 2017. JMC is located in Jimma town, 355 km from Addis Ababa. It is currently the only teaching and specialized hospital in the southwest region of Ethiopia. The hospital serves as a referral site and provides specialized care for Southwest Ethiopia with a catchment population of about 15 million.

### Study population and variables

We included HIV-infected children (< 15 years) who were on ABC and AZT based regimens between October 2014 and April 2017 that fulfill the eligibility criteria. Patients on ABC and AZT based first line regimens, having at least 6 months of follow-up with good adherence, whose records were legible and complete, who have CD4 count at least at base line and 6 months and younger than 15 years, included in the study. Those transferred out within < 6 months of follow up, and patients with incomplete records were excluded. The study was conducted by dividing the total sample in two major classes as ABC group and AZT group.

Data were collected on socio-demographic characteristics (age, sex**,** area of residence, weight (kg), height (cm), body mass index (BMI)), Diseases Related Factors (CD4 count, WHO clinical stage), Treatment Related Factors **(**types of ART regimen, Opportunistic Infection (OI) prophylaxis (Cotrimoxazole Prevention Therapy (CPT), Isoniazid Preventive Therapy (IPT), anti-tubercular drug treatment) and clinical outcomes (mortality, occurrence of OIs).

### Patient enrolment

**Fig. 1:**
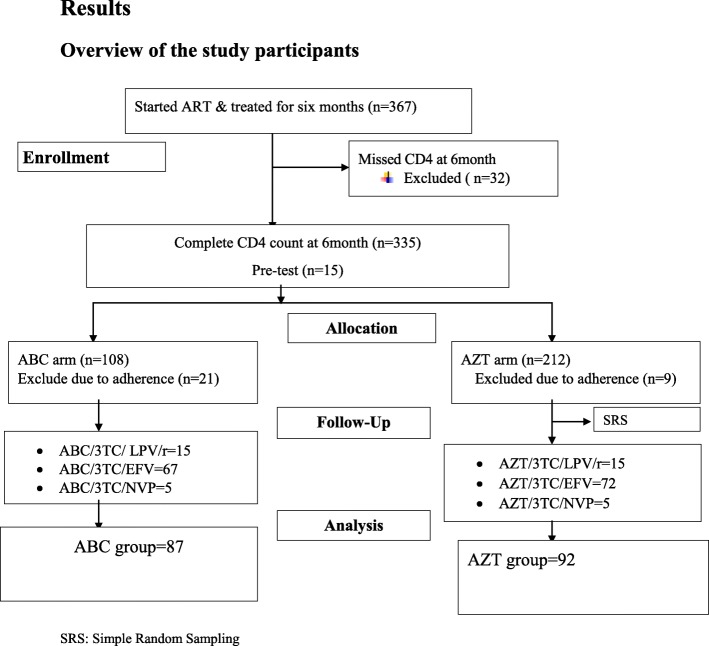
Sample recruitment chart at JMC; of patients attending ART clinic, April 10–May 10, 2017

The number of patient charts who fulfilled the eligibility criteria from ABC group was very limited. i.e. 87 patient charts only. Hence, we included all of them. On the other hand, there were about 203 eligible patient charts from AZT group. Hence, a simple random sampling technique was used to select 92 patient charts from AZT group, making the ABC to AZT group ratio of 1:1.05. Therefore, a total of 179 patient charts, 87 charts from ABC and 92 charts from AZT group were included in the final analysis (Fig. 1).

### Data collection procedure and quality assurance

Data on demographic, clinical, laboratory, and drug administered, were collected by record review using English version checklist. The data collection tool was carefully prepared after reviewing relevant literatures to enable the data collectors to gather all the information required to address the study objectives. A 2-day training was provided on the data collection tool and the general procedures for data collectors i.e. 2 pharmacists (B.Pharm) and 2 nurses, and one medical interns who were acting as supervisor. Pharmacists collected data regarding antiretroviral drugs, and nurses collected patient information from patient chart, ART clinic intake form, and HIV care/ART follow up. Baseline body mass index of the subjects was later calculated after collection of baseline height and weight of the patient from patients chart. Pre-test was conducted on 5% of the eligible records.

### Ethical consideration

The study was approved by Institutional Review Board (IRB) of Jimma University. It has designated with an IRB number of IHRPGB/112/2017. The need for informed consent was waived because of the retrospective, anonymous nature of the study. During data collection, confidentiality was ensured and for this reason, name and address of the patient was not recorded in the data collection check list.

### Statistical analysis

Data were entered into Epi-Data and exported to STATA 13.1 for cleaning and analysis. Descriptive analysis was performed and results were presented by text, tables and charts. Kaplan-Meier (log rank test) was used to compare baseline survival characteristics of the patients for opportunistic infections. Chi-square test was performed to check adequacy of cells before performing Cox regression. Cox regression model assumption of proportional hazards was checked by testing an interaction of covariates with time. Bivariate Cox regression was performed to identify candidate variables for multivariable Cox regressions. Variables with *p*-value ≤0.25 in bivariate regression were considered as candidates for multivariable regression. Multivariable Cox regression was performed to identify independent predictors of opportunistic infections. Hazard ratio with 95% confidence intervals was used as a measure of strength of association and p-value < 0.05 was considered to declare a statistical significance. Finally, a matching estimator, propensity score matching was conducted to estimate the average treatment effect of ABC based regimen on the opportunistic infection considering AZT based regimen as a reference regimen. This is a robust analytical method which uses the idea of randomized clinical trials with an assumption of unconfoundedness. So, it is a better analysis method to show the true result of an intervention in observational studies. It is a matching technique (estimator) that uses the idea of randomized controlled studies in which the impact of confounding variables is minimized.

### Operational definition of terms

**Good adherence**: estimated adherence level of > 95% [22] as recorded by ARTphysicians/Nurses.

**Child:** Age < 15 years [4].

**Incident OIs**: The onset of new infection after 3 months of ART initiation in a patient initially free of any clinically evident infection [23].

**Lost to follow-up:** Refers to a patient who has missed clinical or drug pick-up appointment permanently [24].

## Results

### Overview of the study participants

A total of 367 patients started antiretroviral therapy (ART) and treated for at least 6 months. Of these, 108 from ABC group, and 212 from AZT group have complete CD4+ count at the 6th month of treatment. Thirty two patients were excluded initially from either regimens due to missed CD4+ count at 6 month, 30 (21 and 9 from ABC and AZT respectively) because of adherence problems, and 179 patients were included in the analysis (Fig. 1).

The overall analysis time at risk was 48,330 days. The mean + standard deviation (SD) duration of follow up was 939.8 + 478.3 and 984.92 + 453.1 days for ABC and AZT groups respectively.

### Descriptive analysis of baseline characteristics

Of 179 patients, 98 (54.7%) were females. The mean + SD age of the study participants was 6.6 + 2.8 and 6.5 + 2.9 years for ABC and AZT groups respectively (*p* = 0.25).

At baseline, the mean + SD CD4 T Lymphocyte count was 166.3 + 76.22 and 178.8 + 71.12 cells/mm^3^ for ABC and AZT groups, respectively (*p* = 0.26). The comparative baseline characteristics of the study subjects are depicted in Table 1.

**Table 1:** Comparative baseline characteristics of the cohort at JMC, April 10–May 10, 2017

All *n* = 179		ABC group (*n* = 87)	AZT group (*n* = 92)	*P*-value
Variables				
Sex	Male	42 (48.3%)	39 (42.4%)	0.42
	Female	45 (51.7%)	53 (57.6%)	
Age (years)	< 3 years	11 (12.6%)	12 (13.0%)	0.97
	3–5 years	18 (20.7%)	20 (21.7%)	
	> 5 years	58 (66.7%)	60 (65.2%)	
BMI (baseline)	<5th centile	74 (85.0%)	66 (71.7%)	0.03
	>5th centile	13 (14.9%)	26 (28.3%)	
Maternal HIV status	Positive	78 (89.7%)	83 (90.2%)	0.91
	Unknown	9 (10.3%)	9 (9.8%)	
Area of residence	Urban	66 (75.9%)	68 (73.9%)	0.76
	Rural	21 (24.1%)	24 (26.1%)	
Baseline CD4+ (Mean + SD)		166.31 + 76.22	178.78 + 71.12	0.26
WHO stage	I	8 (9.2%)	3 (3.3%)	0.08
	II	24 (27.6%)	40 (43.5%)	
	III	45 (51.7%)	42 (45.7%)	
	IV	10 (11.5%)	7 (7.6%)	
Functional status	W/A	72 (82.8%)	88 (95.7%)	0.001
	A/D	12 (13.8%)	0 (0.0%)	
	B/R	3 (3.4%)	4 (4.3%)	
TB (treatment)	Yes	3 (3.4%)	9 (9.8%)	0.06
	No	84 (96.6%)	83 (90.2%)	
OI Prophylaxis	Both CPT and INH	85 (97.7%)	89 (96.7%)	0.69
	CPT only	1 (1.1%)	1 (1.1%)	
	Neither	1 (1.1%)	2 (2.2%)	
Nutritional status	Normal	45 (51.7%)	57 (62.0%)	0.17
	SAM	42 (48.3%)	35 (38.0%)	

*BMI* body mass index, *W/A* working/Appropriate, *A/D* Ambulatory/Delay, *B/R* Bedridden/regression, *TB* Tuberculosis, *CPT* cotrimoxazole preventative therapy, *INH* Isoniazid, *CD4* cluster of differentiation, *SD* Standard deviation, *OIs* opportunistic infections

#### Clinical outcomes

##### Mortality

During the study period, a total of 4 patients (1(1.14%) patient from ABC group and 3 (3.3%) from AZT group; *p* = 0.339) died.

##### Opportunistic infections

A total of 58 patients with opportunistic infections were identified, making the overall incidence of opportunistic infection among the cohort 3.9/100,000 person years (py). Most of the OIs were reported from AZT +3TC + EFV (24/71) and ABC +3TC + EFV (23/68). Other regimens contributed as follows: ABC +3TC + Lop/r (5/15), AZT +3TC + Lop/r (5/15) and ABC + 3TC + NVP (1/5). There is no report of OI from AZT + 3TC + NVP regimen. Bacterial pneumonia was reported to be the leading OI (Table 2).

**Table 2:** Opportunistic infections diagnosed in the cohort at JMC, April 10–May 10, 2017

Type of opportunistic infections	Frequency (%)
Bacterial Pneumonia	42 (53.8)
Diarrhea	9 (11.5)
Candidiasis	9 (11.5)
Pneumonia + Pulmonary TB	9 (11.5)
Pneumocystis pneumonia (PCP)	6 (7.7)
Herpes zoster + Candidiasis	6 (7.7)
Pneumonia + Disseminated TB	6 (7.7)
Herpes zoster	6 (7.7)
Pulmonary TB	3 (3.8)

*TB* Tuberculosis*; PCP* Pneumocystis jiroveci Pneumonia

**Fig. 2:**
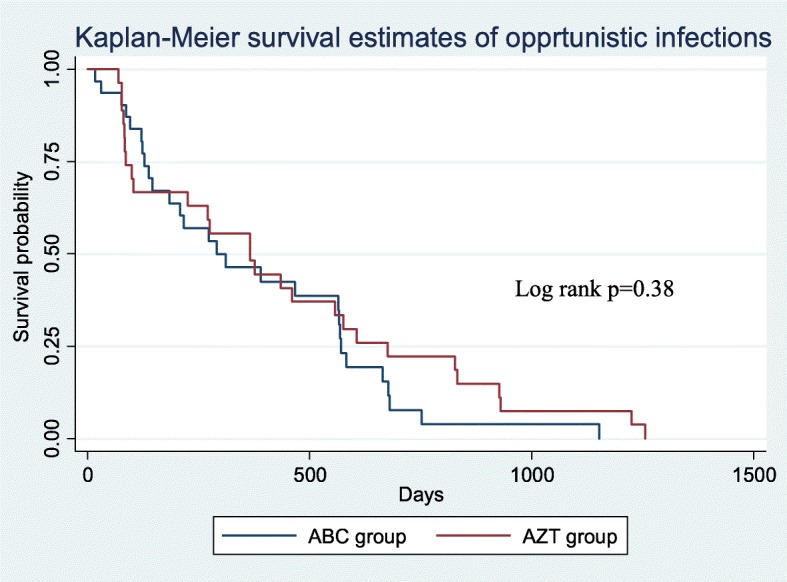
The cumulative survival probability for the occurrence of opportunistic infections among paediatrics exposed to ABC versus AZT in Jimma medical centre, from April 10–May 10, 2017

There were 16/87 from ABC group and 15/92 from AZT cases of pneumonia. The incidence of opportunistic infection attributed to ABC group was 8.77/100,000py] and that of AZT was 6.9/100,000py. The incidence rate ratio (IRR) was found to be 0.87, 95% CI [0.49–1.53], implying that the difference is statistically insignificant (*p* = 0.304). Similarly, the overall median survival time for the cohort was 275 (107–526) days. The median survival time belongs to AZT group was 366 (86–676) days and that of ABC group was 273 (123–569) days (log rank *p* = 0.38). See Fig. 2 below.

### Results of treatment effect analysis

We carried out a robust analysis to estimate the unconfounded effect of ABC based regimen on the occurrence of opportunistic infections both at the population level and among those who received the actual treatment. Because adherence is critical variable, we only included patients with good adherence. In the analysis, the occurrence of opportunistic infections was considered as an outcome dependent variable and treatment group was considered as treatment dependent variable. After adjusting for sex, age, base line body mass index, weight for height, in care of the child, maternal status, paternal status, residence, baseline CD4 count, viral load, WHO Stage, nutritional status, and exposure to anti-tubercular drugs, the propensity score matching analysis failed to show the superiority of ABC based regimen in suppressing OI at both population level (β = 0.0112**,** *p* = 0.844) and among those who received the actual treatment (β = 0.0217, *p* = 0.772) (Table 3). Although it seems there was 22 excess opportunistic infections in ABC group among 1000 patients who received the actual therapy, the finding is statistically insignificant (p = 0.772) (Table 3). This section of analysis was also in line with the Kaplan-Mayer survival analysis output shown in Fig. 2 above.

**Table 3:** The estimated average treatment effect of ABC based regimen on opportunistic infections at JMC

Treatment group		Coefficient (β)	AI Robust Standard Errors	p	95% CI
AZT		Base Regimen			
ABC	ATE	0.0112	0.0566	0.844	−0.0998, 0.1221
	ATET	0.0217	0.0750	0.772	−0.1253, 0.1688

*ATE* Estimated average treatment effect in the population, *ATET* Estimated average treatment among treated, *CI* Confidence interval, *AI* Abadie-imbens

### Predictors of opportunistic infections

After checking for model fitness and considering all the assumptions, a Cox-proportional hazard regression was conducted to identify predictors for the development of OIs. On bivariate Cox-regression, baseline body mass index, baseline weight for height (WFH), area of residence, baseline CD4 count, baseline viral load, WHO clinical stages, nutritional status and exposure to anti-tubercular drugs were associated with the development of OIs. After adjusting all the confounders with Cox-proportional hazard model, baseline CD4 count, exposure to anti-tubercular drugs and nutritional status remained independent predictors of OIs. Accordingly, there was a 1% reduction in the hazard of OIs development as median baseline CD4 count increased by a unit (AHR = 0.99, 95% CI [0.98–0.99]. However, hazard of OIs in paediatric patients with malnutrition is extremely high (AHR = 15.92 [5.34–47.50]). Moreover, exposure to tuberculosis treatment had also carried significant risk of OIs, (AHR = 2.93 [1.39–6.17]) (Table 4). Functional status and OIs prophylaxis were removed from the analysis, as they did not pass the cell adequacy test.

**Table 4:** Crude and adjusted Cox-proportional hazard regression output for the predictors of opportunistic infections among HIV -infected paediatrics at JMC, April 10–May 10, 2017

Variables		OIs		CHR [95%CI]	*p*-value	AHR[95%CI]	*p*-value
		Yes	No				
Sex	Male	30 (30.6	68 (69.4)	1			
	Female	28 (34.6)	53 (65.4)	0.94 [0.55–1.59]	0.810		
Age: Median IQR	7 (4–9)	58 (32.4)	121 (67.6)	1.00 [0.91–1.09]	0.995		
Base-line body mass index	Below 5th centile	53 (37.9)	87 (62.1)	3.63 [1.45–9.10]	0.006		
	Above 5^th^centile	5 (12.8)	34 (87.2)	1			
Weight for height	< 70%	15 (41.7)	21 (58.3)	1.34 [0.74–2.44]	0.332		
	70–85%	2 (14.3)	12 (85.7)	0.42 [0.10–1.73]	0.229		
	> 85%	41 (31.8)	88 (68.2)	1			
In care of the child	Mother	7 (29.2)	17 (70.8)	1			
	Other	51 (32.9)	104 (67.1)	0.66 [0.28–1.54]	0.332		
Maternal status	Live	47 (32.2)	99 (67.8)	1			
	Dead	11 (33.3)	22 (66.7)	1.17 [0.59–2.32]	0.654		
Maternal sero-status	Unknown	6 (33.3)	12 (66.7)	0.71 [0.28–1.81]	0.476		
	Negative	52 (32.3)	109 (67.7)	1			
Paternal status	Live	43 (32.6)	89 (67.4)	1			
	Dead	15 (31.9)	32 (68.1)	0.88 [0.48–1.62]	0.694		
Residence	Urban	49 (36.6)	85 (63.4)	1			
	Rural	9 (20)	36 (80)	1.81 [0.885–3.69]	0.104		
ART group	ABC	27 (31)	60 (69)	1.05 [0.62–1.76]	0.885		
	AZT	29 (31.5)	63 (68.5)	1			
CD4 count^a^	162 (117–221)	58 (32.4)	121 (67.6)	0.98 [0.97–0.98]	< 0.001	0.99 [0.98–0.99]	< 0.001
Baseline Viral load	< 1000copes/ml	36 (24.7)	110 (75.3)	1		1	
	>1000copes/ml	19 (65.5)	10 (34.5)	2.87 [1.62–5.10]	< 0.001	1.72 [0.91–3.24]	0.094
WHO Stage	Stage I	5 (45.5)	6 (54.5)	1			
	Stage II	15 (23.4)	49 (76.6)	0.39 [0.14–1.06]	0.066		
	Stage III	31 (35.6)	56 (64.4)	0.59 [0.23–1.52]	0.276		
	Stage IV	7 (41.2)	10 (58.8)	0.80 [0.25–2.53]	0.707		
Baseline nutritional status	Normal	4 (3.9)	98 (96.1)	1		1	
	SAM	54 (70.1)	23 (29.9)	28.37 [10.23–78.73]	< 0.001	15.92 [5.34–47.50]	< 0.001
TB treatment	No	46 (27.7)	120 (72.3)	1		1	
	Yes	12 (92.3)	1 (7.7)	8.58 [4.33–17.01]	< 0.001	2.93 [1.39–6.17]	0.005

*CHR* Crude hazard ratio, *AHR* Adjusted hazard ratio, *ABC* Abacavir, *ART* antiretroviral therapy, *AZT* Azidothymidine, *WHO* world health organization, *TB* Tuberculosis

^a^Baseline CD4 count expressed in median and interquartile range (IQR)

## Discussion

This is the first study to compare the clinical outcomes of ABC and AZT based regimens among HIV-infected children in Ethiopia. Our analysis showed that overall there was no difference in mortality and occurrence of OIs between children receiving ABC or AZT based ART in JMC, Ethiopia (Fig. 2, Tables 3 and 4).

Baseline CD4 count, nutritional status, and exposure to tuberculosis treatment were the independent predictors for the development of OIs.

The reported mortality between the ABC and AZT groups was statistically insignificant. There was only 1 (1.14%) death from ABC group and 3 (3.3%) deaths from AZT group (*p* = 0.339). Similarly, there was no difference in the incidence of opportunistic infection (IRR = 0.87, 95% CI [0.49–1.53, *p* = 0.304). Moreover, the finding from propensity score matching showed statistically insignificant difference of ABC based regimen’s effect on opportunistic infections (β = 0.0217, *p* = 0.772). A similar finding was obtained in the CHAPAS-3 study. This RCT compared the NRTIs ABC, AZT and d4T in HIV-infected African children, and found that all three NRTIs had similar outcomes in terms of clinical, immunological and virological responses [11]. A finding by Adetokunboh et al. also concluded that ABC based regimen in children had similar efficacy and safety profile with AZT based regimens [25]. In contrary, the PENTA-5 trial demonstrated greater efficacy of ABC based regimen over AZT [18]. This difference could be attributed to study design, sample size (179 vs.128 subjects), inclusion of patients only with good adherence, and exclusion of patients with follow-up less than 6 months.

This study showed that baseline CD4 count was strongly associated with the development of OIs. Accordingly, there was a 1% reduction in the hazard of OIs development as median baseline CD4 count increased by a unit (AHR = 0.99, 95% CI [0.98–0.99]). Similar findings were found from Ethiopia [26] and Nigeria [27].

However, paediatric patients with malnutrition had extremely high risk of having opportunistic infections (AHR = 15.92, 95% CI [5.34–47.50]), similar to studies in Ethiopia [28], Nigeria [27] and Cambodia [29]. Malnutrition aggravates the underlying immunosuppression enhancing their susceptibility to various opportunistic infections and HIV disease progression [30, 31].

Moreover, exposure to tuberculosis treatment had also carried significant risk of OIs. Accordingly, patients with anti-tubercular drug treatment were three times at higher risk of having opportunistic infections (AHR = 2.93, 95% CI [1.39–6.17]). This might be related to the killing of mycobacteria by anti-tubercular drugs releasing large amounts of mycobacterial antigens, which can stimulate an exuberant inflammatory response. The resulting excessive inflammatory response leads to unmasking of an underlying occult opportunistic infections [32].

The results of our study should be interpreted in the context of several possible limitations. Firstly, as the sample size was relatively small the power to detect definitive differences may have been limited. Measure of adherence by health professionals that may not fit to the reality, and inability to assess the occurrence of specific OIs are some of the limitations.

## Conclusion

In present study, there was no statistically significant difference in mortality and occurrence of opportunistic infections between those exposed to ABC versus AZT based regimens. Baseline CD4 count, nutritional status, and exposure to tuberculosis treatment were the independent predictors for the development of OIs. The study highlighted the need for special attention for these patients groups over the course of treatment provision.

## Data Availability

The datasets and materials used in our study are available from the corresponding author on reasonable request.

## Abbreviations

AHR: Adjusted Hazard Ratio
ART: Antiretroviral Therapy
CHR: Crude Hazard Ratio
CI: Confidence interval
HIV: Human Immune Virus
JMC: Jimma Medical Center
OIs: Opportunistic Infections
